# Indian-Ink Perfusion Based Method for Reconstructing Continuous Vascular Networks in Whole Mouse Brain

**DOI:** 10.1371/journal.pone.0088067

**Published:** 2014-01-30

**Authors:** Songchao Xue, Hui Gong, Tao Jiang, Weihua Luo, Yuanzheng Meng, Qian Liu, Shangbin Chen, Anan Li

**Affiliations:** 1 Britton Chance Center for Biomedical Photonics, Huazhong University of Science and Technology-Wuhan National Laboratory for Optoelectronics, Wuhan, China; 2 MoE Key Laboratory for Biomedical Photonics, Department of Biomedical Engineering, Huazhong University of Science and Technology, Wuhan, China; Katholieke Universiteit Leuven, Belgium

## Abstract

The topology of the cerebral vasculature, which is the energy transport corridor of the brain, can be used to study cerebral circulatory pathways. Limited by the restrictions of the vascular markers and imaging methods, studies on cerebral vascular structure now mainly focus on either observation of the macro vessels in a whole brain or imaging of the micro vessels in a small region. Simultaneous vascular studies of arteries, veins and capillaries have not been achieved in the whole brain of mammals. Here, we have combined the improved gelatin-Indian ink vessel perfusion process with Micro-Optical Sectioning Tomography for imaging the vessel network of an entire mouse brain. With 17 days of work, an integral dataset for the entire cerebral vessels was acquired. The voxel resolution is 0.35×0.4×2.0 µm^3^ for the whole brain. Besides the observations of fine and complex vascular networks in the reconstructed slices and entire brain views, a representative continuous vascular tracking has been demonstrated in the deep thalamus. This study provided an effective method for studying the entire macro and micro vascular networks of mouse brain simultaneously.

## Introduction

The topology of the cerebral vasculature, which is the energy transport corridor of the brain, can be used to study cerebral circulatory pathways [1]–[3]. All arteries, veins and capillaries work together to meet the demand for an uninterrupted energy supply for the brain. The macro vessels were identified first and were studied with the naked eye. The Micro-CT technique, which has been used in mice, can visualize the arterioles and venules in the whole brain. Dorr et al. identified and marked the major vessels in the CBA mouse using Microfil perfusion and Micro-CT imaging [4], [5]. MRI techniques can also visualize the macro vessels in the whole mouse brain [6], [7]. To observe the smaller, more complex micro vessels, *ex vivo* two-photon laser scanning microscopy is used to image the capillary network in the cortex to a depth of 1 mm using fluorescent gelatin vessel perfusion [8].

However, current cerebrovascular studies focus mainly either on the macro vessels of the whole brain or on the micro vessels in a small local field separately. The existing techniques used to observe the macro vessels cannot visualize the fine branches of the veins, arteries and the capillary network connecting them. On the other hand, microvascular imaging in a small field cannot obtain a clear view of the vessels' origins and destinations.

Cerebral vessels play significant roles in the development and degradation of the neural network and in the process of maintaining normal brain functions [9], [10]. This leads to an urgent need for systematic research. In recent years, the study of the vascular network has become a hotspot [8], [11]. Several technologies have been developed to attempt to bridge the macro and micro vessels which form the continuous vascular network. Vascular casts and integrated μCT, SRμ-CT and SEM have been used to image the cerebral vasculature at different resolutions [12]. Optical microscopes with three-dimensional scanning and stitching have also been used in preliminary experiments to obtain cerebrovascular dataset [13]–[16], some resource have been available on the internet (http://kesm.cs.tamu.edu/index.php?select=v1). However, reconstructing the connections of the blood supply pathway from the feeding vessels to the draining vessels through the capillaries in the whole brain range, i.e. cerebrovascular connection, has not been achieved yet.

To study the entire macro and micro vascular anatomical structure network, we improved the gelatin-Indian ink vessel perfusion process based on previous work [4], [5], [8], [13], [17], [18] and obtained a desirable perfused Kunming mouse brain. After embedding the brain with Spurr resin, a complete three-dimensional cerebrovascular dataset was acquired using Micro-Optical Sectioning Tomography (MOST) [19]. Based on the dataset, the thalamus was selected as an example, and its blood supply pathway was tracked and studied in this paper.

## Materials and Methods

All 9 eight-week-old healthy Kunming mice (Hubei Province Experimental Animal Research Center, China) were used in this study (8 for ink perfusion, 1 for two-photon imaging). Animal care and use was performed in accordance with the guidelines of the Administration Committee of Affairs Concerning Experimental Animals in Hubei Province, China. The protocol was approved by the Committee on the Ethics of Animal Experiments of the Huazhong University of Science and Technology (Permit Number: 00027467). All surgery was performed under anesthesia, and all efforts were made to minimize suffering.

### Indian-ink perfusion

Except for 7 mice used in the preliminary perfusion experiments, one Kunming mouse was used in the optimized Indian-ink perfusion process. The mouse was anesthetized with an intraperitoneal injection of 5% chloral hydrate (1 mL/100 g). We opened the chest after the mouse had been completely anesthetized. A syringe needle (Φ = 1.6 mm) was then inserted into the left ventricle, and a small slit was cut in the right atrium. Phosphate buffered saline (PBS, 0.01 mol/L, pH 7.2–7.4, preheated to 37°C) was perfused at 40–60 mmHg at a filling rate of 3–5 mL/min through the aorta until the blood outflow from the right atrium stopped. The perfusion process typically lasted for 1–2 min and was followed by perfusion with a 10% formalin solution (containing 0.01 mol/L PBS, pH 7.2–7.4, preheated to 37°C) for 3–5 min using the same perfusion parameters. Then, 10 mL 10% India ink mixed with 2% gelatin (preheated to 40°C) was injected manually using a 20-mL disposable medical syringe at the rate of 3–5 mL/min. The component of 2% gelatin has been reported be helpful for complete vascular perfusion [8]. After injection, the mouse was placed ventral side up at −20°C for 10–20 min until the gelatin solidified. We then cut the head and carefully removed the entire mouse brain. With visual inspection, a black and uniform brain surface indicates effective perfusion of the brain (refer to Figure S1). The preliminary Indian-ink perfusion experiments revealed that the temperature and the perfusion rate and pressure are very important in obtaining desirable whole-brain perfusion. There were three key points of the whole improved Indian-ink perfusion procedure:

1. All PBS and formalin solutions were filtered through a 0.8 µm nylon membrane and hung 1.5 meters (i.e. 110 mmHg) above the operating plane to provide the suitable perfusion pressure.
2. At the operating plane, a mercury sphygmomanometer was connected to the syringe needle using a three-way valve to measure the real pressure in the aorta, and the pressure was controlled by adjusting the perfusion rate using a limiting valve.
3. India ink (10%) mixed with 2% gelatin was prepared as follows: a 250 mL Erlenmeyer flask was filled with 176 g deionized water, 4 g gelatin (no. G1890, Sigma-Aldrich, St. Louis, MO, USA) and a clean magnetic stir bar, then covered with sealing film to exclude dust. After heating in a water bath on a magnetic stirrer at 60°C for one hour (until the gelatin was completely dissolved), the temperature was changed to 40°C. Finally, 20 g India ink was added, and the solution was stirred for 24 hours.

### Data acquisition

The optimally perfused mouse brain was imaged using a home-made Micro-CT [20] to measure the original size of the brain without physical sectioning. The voltage used was 65 KV, the power was 35 W, and the three-dimensional reconstruction voxel size for the Micro-CT was 31.9×31.9×31.9 µm^3^. Finally, the brain was bathed in a 10% formalin solution (containing 0.01 mol/L PBS, pH 7.2–7.4, 4°C) for one week.

In order to employ MOST system to get high-resolution images, the Indian-ink perfused brain should be dehydrated and embedded following the work of Zhang et al [21]. Briefly, the brain was dehydrated with ethanol and acetone sequentially for 24 hours, infiltrated in graded series of Spurr resin solution (SPI, USA) for 32 hours, then embedded in 100% Spurr resin solution and solidified for 36 hours in an oven at 60°C. The embedded mouse brain was mounted into MOST system for automatic data acquisition [19]. The whole mouse brain were sectioned with 2 µm layer interval and imaged on the coronal plane from the rostral end to the caudal end. A 40X water immersion objective (NA = 0.8) and a time-delay integration line-scan charge-coupled device (TDI-CCD) were used in MOST system [19]. The ultimate voxel size of MOST is 0.35×0.4×2 µm^3^. The data acquisition was uninterrupted and lasted for about 5 days. All the image tiles were saved in 8 bit depth.

### Image processing

A graphic workstation (CPU Intel Xeon E5-2690, NVIDIA Q6000, 96 GB RAM) was used to for image processing. Firstly, a customized MATLAB (Mathworks, Inc.) program was used to stich the image tiles to form coronal slices automatically. The original image dataset consists of over 7000 image slices. To enhance vessels observation, the image slices were performed with intensity inverting and contrast correction using ImageJ software (http://rsb.info.nih.gov/ij/). For easier handling, the original image dataset was resampled into two derived datasets with resolutions of 1×1×2 µm^3^ and 4×4×4 µm^3^. The former dataset was used to display the capillaries in a local field, while the latter was used to display the entire dataset and to perform image dataset registration. In addition, the original vessels were interpolated to 0.7×0.7×1.0 µm^3^ and 0.2×0.2×0.2 µm^3^ with cubic spline for tracking the accurate diameters of the vessels using the third-party software NeuronStudio [22] and Amira, respectively. Although the interpolation operation increases the computational task, it led to an improvement in accuracy while using these two software programs.

#### MIP reconstruction of the brain

For the 1×1×2 µm^3^ dataset, five 100 µm thick coronal planes were selected and reconstructed using maximum intensity projection (MIP) in the olfactory bulb, frontal cortex, hippocampus, midbrain and cerebellum to evaluate the perfusion performance. To visualize the macro vessels in the whole brain, the 4×4×4 µm^3^ dataset was low-pass filtered using a Gaussian sphere with a diameter of 14 µm, thus reducing the intensity of the smaller vessels and the noise. The larger vessels were shown and marked according to the cerebrovascular atlas [5].

#### Measurement of capillary diameter

14 regions of interest (ROIs) at 400-cubic-micron were manually selected in the brain and tracked using the Amira program to obtain the distribution of vascular calibers. To measure the shrinkage of the mouse brain caused by the dehydration and Spurr resin embedding processes, we applied the affine registration in Amira with a cross-correlation algorithm to register the Micro-CT data and the 4×4×4 µm^3^ cerebrovascular dataset. The shrinkage was defined as the ratio of reconstructed three-dimensional MOST image (present images) to Micro-CT images (reflecting normal shape brain) in three main axes (Anterior-Posterior, Left-Right, Ventral-Dorsal). The resultant shrinkage factors were used to recover the diameter of vessels measured from the MOST dataset.

#### Continuous vessel tracking in thalamus

Due to the importance in sensory sensing and stroke plasticity, the thalamus was selected to study its blood supply [23]. Based on the cerebrovascular atlas of the CBA mouse and the brain partition maps in C57 mice [24], we identified and located the vessels and regions in the dataset using vascular landmarks [4]. In the 4×4×4 µm^3^ cerebrovascular dataset, some particular regions were segmented manually and reconstructed with a surface view using Amira segmentation tools, including the vertebral artery (VA), basilar artery (BA), superior cerebellar artery (SCA), posterior cerebral artery (PCA), thalamoperforating artery, posterior communicating artery (PcomA), internal carotid artery (IC), anterior cerebral artery (ACA), middle cerebral artery (MCA), azygos of the anterior cerebral artery (azACA), thalamostriate vein, great cerebral vein of Galen and transverse sinus. In the 0.7×0.7×0.7 µm^3^ cerebrovascular dataset, the voxel data containing the right thalamus was cut to a box of 2.5×2.3×2.9 mm^3^, and the vessels were tracked automatically using NeuronStudio. With the help of the Filament Editor tool in Amira, some gaps that were caused by NeuronStudio, could be connected manually. The tracking process was shown in the Supporting Information as Video S1.

## Results

**Figure 1:**
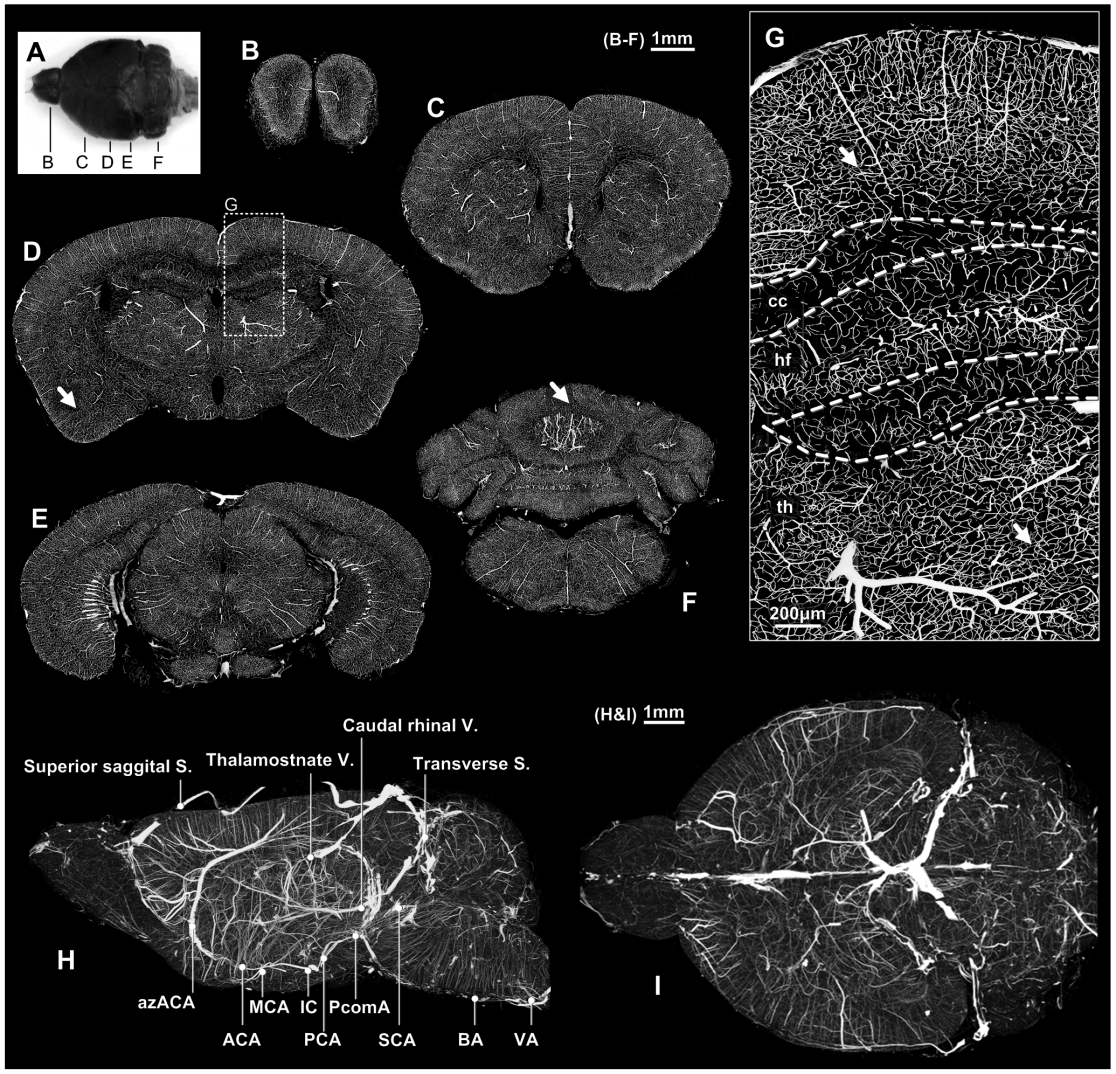
Vessel acquisition and reconstruction in the whole mouse brain. A: The dorsal view of the gelatin-Indian ink perfused brain; after embedding with Spurr resin and imaging using MOST to acquire the three-dimensional cerebrovascular dataset, five 100-µm thick coronal planes were selected and MIP-reconstructed in the olfactory bulb (B), frontal (C), hippocampus (D), midbrain (E) and cerebellum (F); the results showed desirable perfusion in the whole brain, and no blank areas were found in the brain entity. G: ROI in Figure 1D; the capillary network was continuous, and no gaps were found. H: The left view of the whole brain; several major vessels were marked. I: The top view of the whole brain. cc: corpus callosum; hf: hippocampus; th: thalamus. B–F: Bar = 1 mm; G: Bar = 200 µm, H: Bar = 1 mm.

For Indian-ink perfusion, a desirable even filling of the mouse brain was judged by black and uniform color on the surface (Figure 1A, dorsal view). The success rate to achieve proper perfusion for the method was 1–2 out of 8 mice. It took about 17 days from Indian-ink perfusion to MOST data acquisition (image tiles) and an integral dataset for the entire cerebral vessels was acquired in this study. In addition, three weeks were needed for the image processing involved in this study. The raw MOST dataset of the perfused mouse brain was about 3 TB. The registration process between the CT data and the MOST data showed that the mouse brain had experienced near isotropic shrinkage: Anterior-Posterior, 83.7%; Left-Right, 83.4%; Ventral-Dorsal, 84.1%. An average value of 83.7% was used to revise the vessel diameter.

### Overview of vascular networks

With MIP reconstruction, this study presented a general view of the vascular structure in coronal slices (Figure 1B–F). The whole brain entity was perfused with enormous visible vessels, and no blank areas were found (Video S2,S3). From an enlarged view (Figure 1G), the vascular networks at different depth seem like continuous, and no incomplete perfusion areas are found with visual inspection. Using a low-pass filter, the large vessels became visible in the left and dorsal view of the whole brain (Figure 1H, I). Refer to the cerebrovascular atlas [5], all major vessels were found and marked in Figure 1H. An average of 14 measurements of selected capillaries in the abovementioned 14 ROIs indicated the caliber is 3.9±1.0 µm. Taking the shrinkage into consideration, the original capillary calibers without shrinkage should be about 4.6±1.2 µm. In supplementary experiment, we test the accuracy of MOST measurements on vessels by comparing with *in vivo* two-photon imaging. Both 30 loci measurements from barrel cortex showed no significant difference (4.3±0.6 µm vs. 4.6±1.1 µm, *p* = 0.2; details in Figure S2).

### Continuity of vascular network

**Figure 2:**
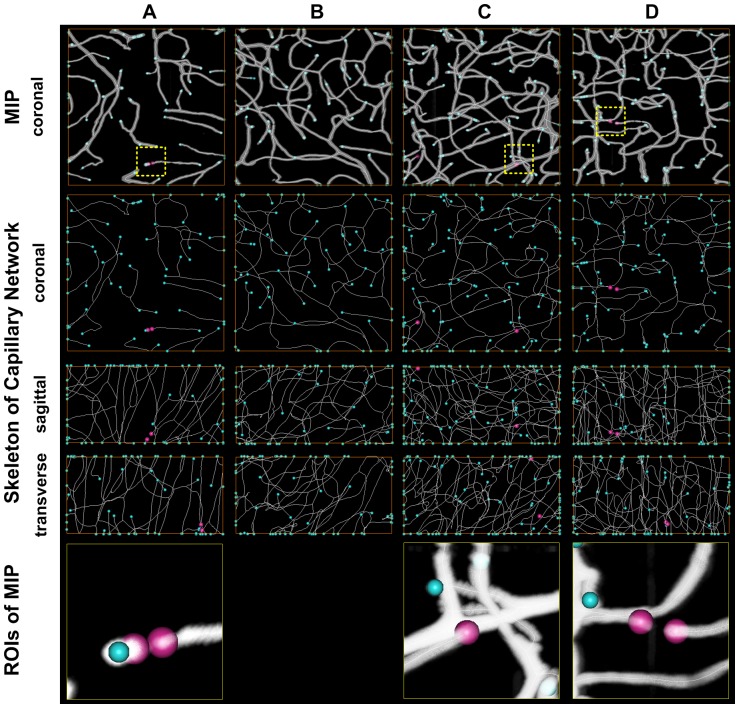
Assessment of capillary continuity. Four ROIs (Columns A–D) with size of 200×200×100 µm^3^ at cortex, amygdaloid nucleus, thalamus, cerebellar lobule were selected and used to analyze the vascular continuity. The four ROIs were indicated sequentially with white arrows in Figure 1D, F and G. ROI A was taken as an example. On the top row, the MIP reconstruction of this ROI covered with vascular tracking results by using Amira was shown. The vascular terminal could be located through vascular tracking results projection on xy, yz and xz orthogonal planes (Rows 2–4). The vascular terminals inside the ROI were marked with red balls, while the terminals on the boundary were marked with blue balls. The panels on the bottom showed the details around the vascular terminals, most of which were caused by caliber narrowing.

To check the continuity of vascular network in our dataset, 4 ROIs were selected to analyze the vascular continuity in cortex, amygdaloid nucleus, thalamus and cerebellar lobule, each with size of 200×200×100 µm^3^ (Figure 2A–D). The MIP reconstruction of the four ROIs covered with vascular tracking results by using Amira was shown on the top row of Figure 2. The vascular terminal was located through vascular tracking results projection on xy, yz and xz orthogonal planes (rows 2–4 in Figure 2). The vascular terminals inside the ROI were marked with pink balls, while the terminals on the boundary were marked with blue balls. No more than 2 end points (i.e. gap) in each of the 200×200×100 µm^3^ size ROI. With the closer view of the pink terminals, they were found mainly caused by caliber narrowing (the bottom row of Figure 2).

### Vascular network in thalamus

**Figure 3:**
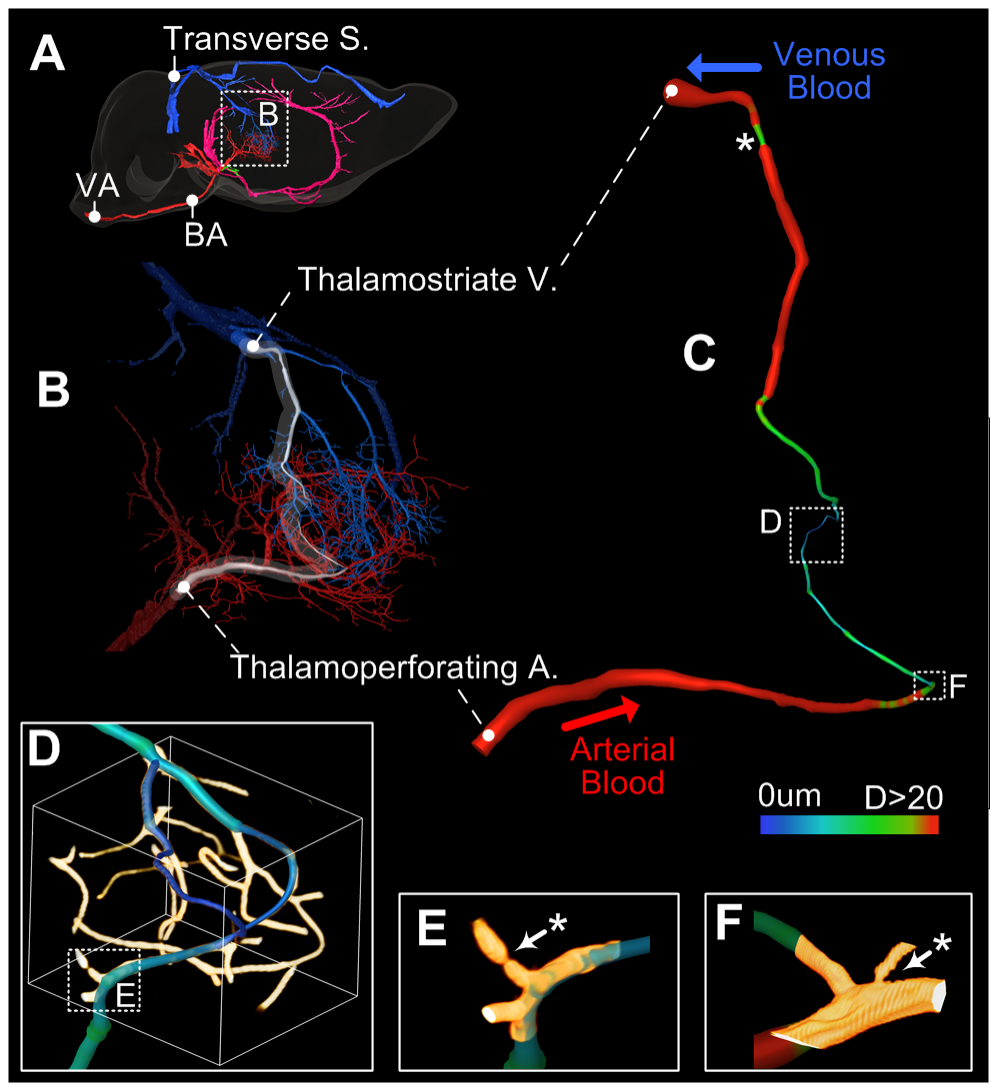
Blood supply pathway tracking and analysis in the right thalamus. A: The right view of the brain. A clear blood pathway begins in the VA, passes through the BA and the thalamoperforating artery, enters the thalamus, and finally drains out through the thalamostriate vein, the great cerebral vein of Galen and the transverse sinus. B: The vessels with diameters larger than 5 µm in the thalamus, red for arteries and blue for veins. C: A vascular segment from the thalamoperforating artery to the thalamostriate vein through the capillary, colored by diameter. D: The capillaries in the pathway from the artery to the vein. E–F: ROIs from D and C, showing the narrowing in the capillaries. * Identifies the vascular narrowing sites in C, E and F.

A representative vascular network was demonstrated in the deep brain region thalamus (Figure 3). Figure 3A shows the blood supply pathway in the thalamus; the blood enters from the VA in the spine, passes through the BA, and enters the thalamus through the thalamoperforating artery at the hypothalamus; after passing through the capillaries, the blood drains through the thalamostriate vein, the great cerebral vein of Galen and the transverse sinus. Figure 3B shows the vessels in the thalamus with diameters larger than 5 µm, which were identified and colored according to their sources (red for arteries and blue for veins). The cerebrovascular branches are tree-like and easily observed. Figure 3C shows a vascular segment from the thalamoperforating artery to the thalamostriate vein through the capillary. Figure three-dimensional shows the capillaries in the pathway from the artery to the vein. The capillaries form a cross-linked interpenetrating network to serve the energy demand of each brain cell. As the vessels begin to enter the capillary at the micro-arterial branch, the calibers suddenly narrow (Figure 3E–F, Figure S4). This sudden caliber narrowing occurs not only in the capillaries but also in the most penetrating arteries and in several penetrating veins (Figure 3C). The asterisks mark the sites of vascular narrowing. More results were shown in Figure S3.

## Discussion

This study established a method based on gelatin-Indian ink perfusion and MOST imaging to simultaneously acquire the entire macro- and microvascular anatomical structures in a mouse brain. This dataset reconstructed most blood vessels under the pia mater, including the arteries, veins and capillaries. In the cerebrovascular dataset, the vessels were desirable perfused in the whole brain and had a significant view compared with the surrounding brain tissue, and the vascular networks were continuous. The incomplete perfusion and the disrupted view of the vessels in the vessel-dense region, which always occur with ink perfusion [13], [14], [18], do not appear in this dataset. Comparing with some similar studies [8], [11], [13], our work presented the two features: the improved resolution and the entire brain scale. Thus, this study provided an important method to image whole mouse brain vasculature in high resolution.

Reconstruction of the macro vessels showed that the major vessels could be located, and their distribution is consistent with the existing cerebrovascular maps [5]. The estimated average diameter of the capillaries is 4.6±1.2 µm with the brain contraction correction, and this result is consistent with the diameter of 4 µm previously reported *in vivo* [8], [11]. The narrowing of the cerebral vascular caliber in the capillary usually occurs at the sub-branches from the large blood vessels, while the narrowing of the cerebral vascular caliber in the penetrating vessels usually occurs at the inlet and the outlet. These results are similar to previous SEM results [25], [26], and these narrowing sites coincide with the distribution of the cerebral blood flow regulation sites.

The blood supply pathway tracking and vascular reconstruction with diameters larger than 5 µm in the thalamus showed more details in the cerebrovascular branches than that could be obtained with Micro-CT or MRI. Reconstruction of the capillaries showed the same details of the capillary networks that have been observed using an optical microscope. Vascular segment tracking and analysis from the thalamoperforating artery to the thalamostriate vein achieved the goal of continuous cerebral vascular visualization from the artery to the vein through the capillaries. We have reconciled the difficult question that vascular imaging in the whole brain cannot observe the capillaries while capillaries imaging is inconvenient to track the whereabouts of their feeding and draining vessels. This work has provided a useful tool for studying the blood supply network of the small mammalian brain and could facilitate the work of cerebrovascular reconstruction, helping the simulation of stroke and the building of a cerebral blood flow model for fMRI.

The presented method to obtain cerebrovascular data was an *ex vivo* method. Although our results cannot reveal the true cerebrovascular state *in vivo*, this method can still provide meaningful information for the structure of vascular network. Due to technique limitation, only the blood vessels in the mouse brain were reconstructed in the present work; other objects, such as neurons, could not be detected. Dual staining may help to obtain images of both the vessels and the neurons simultaneously in future studies. Because of the lack of available automated vascular analysis tools for large amounts of vessel data, only the right thalamus was studied. A platform for tracking and analyzing the large number of vessels in the brain automatically would be helpful for further research on the structural characteristics of the blood supply network in the brain. At last, as data acquisition and processing cost a lot of resources, only one dataset was acquired in this study, as well as the problem how to share the massive original over 3 TB dataset. While the 50, 100 um MIP reconstructions on coronal plane and the 100 um MIP reconstructions on sagittal plane for the whole brain are available at the website (http://bmp.hust.edu.cn/most).

## Supporting Information

Figure S1 **Visual inspection of Indian-ink perfused mouse brain.** A: an effective Indian-ink perfused mouse brain shows uniform and black surface, which is used to get results in this paper; B: an incomplete Indian-ink perfused mouse brain, which is due to the high temperature and perfusion pressure; C: a uniform but incomplete perfused mouse brain, due to the low temperature and perfusion pressure; D: an un-uniform and incomplete perfused mouse brain, due to the impurities in perfusion fluid. Scale bar = 5 mm.
(TIF)

Figure S2 **Comparisons between MOST imaging and** ***in vivo*** **two-photon imaging on calibers measurement.** A: a 2 µm thickness image frame from *in vivo* two-photon fluorescence imaging on barrel cortex in an anesthetized Kunming mouse to measure the capillaries' calibers; B: a 2 µm thickness image frame from the MOST dataset (with intensity reverting and cut from the same dataset acquired in this study); C: 100 µm depth MIP reconstruction of two-photon vascular imaging; D: 100 µm depth MIP reconstruction of MOST vascular imaging. By randomly selecting 30 loci to measure the capillaries calibers in C and D, the two measurements showed consistent results.
(TIF)

Figure S3 **Vessel reconstruction in the cortex, hippocampus and choroid.** A: The locations of the three ROIs. B: Vessel reconstruction and diameter tracking in the cortex using Amira, 1.0×1.0×0.2 mm^3^. C: Vessel reconstruction in the hippocampus, 1.5×1.0×1.5 mm^3^, red for arteries and blue for veins. D: Vessel surface reconstruction of the choroid in the lateral ventricle, 1.0×1.5×1.0 mm^3^; the arrows show the direction of the blood flow.
(TIF)

Figure S4 **The RAW reconstruction of the vessel narrowing site which showed in** **Figure 3** **.** A B C, the right view; D the rear view; C D, volume rendering reconstruction of the vessels around the narrowing site.
(TIF)

Video S1 **Blood supply pathway tracking in the thalamus.**
(MP4)

Video S2 **Slices appear in a sequential way to show 100 um MIP reconstructions on coronal plane for the whole brain.**
(MP4)

Video S3 **Slices appear in a sequential way to show 100 um MIP reconstructions on sagittal plane for the whole brain.**
(MP4)

## References

[1] Shih AY, Driscoll JD, Drew PJ, Nishimura N, Schaffer CB, et al (2012) Two-photon microscopy as a tool to study blood flow and neurovascular coupling in the rodent brain. J Cerebr Blood F Met 32: 1277–1309. 10.1038/jcbfm.2011.196 PMC3390800 22293983

[2] Iadecola C, Nedergaard M (2007) Glial regulation of the cerebral microvasculature. Nat Neurosci 10: 1369–1376. 17965657 10.1038/nn2003

[3] Farkas E, Luiten PG (2001) Cerebral microvascular pathology in aging and Alzheimer's disease. Prog Neurobiol 64: 575–611. 11311463 10.1016/s0301-0082(00)00068-x

[4] Chugh BP, Lerch JP, Yu LX, Pienkowski M, Harrison RV, et al (2009) Measurement of cerebral blood volume in mouse brain regions using micro-computed tomography. Neuroimage 47: 1312–1318. 19362597 10.1016/j.neuroimage.2009.03.083

[5] Dorr A, Sled JG, Kabani N (2007) Three-dimensional cerebral vasculature of the CBA mouse brain: a magnetic resonance imaging and micro computed tomography study. Neuroimage 35: 1409–1423. 17369055 10.1016/j.neuroimage.2006.12.040

[6] Klohs J, Baltes C, Princz-Kranz F, Ratering D, Nitsch RM, et al (2012) Contrast-Enhanced Magnetic Resonance Microangiography Reveals Remodeling of the Cerebral Microvasculature in Transgenic ArcA beta Mice. J Neurosci 32: 1705–1713. 22302811 10.1523/JNEUROSCI.5626-11.2012 PMC6703349

[7] Pathak AP, Kim E, Zhang JY, Jones MV (2011) Three-Dimensional Imaging of the Mouse Neurovasculature with Magnetic Resonance Microscopy. PLoS One 6: e22643. 21818357 10.1371/journal.pone.0022643 PMC3144917

[8] Tsai PS, Kaufhold JP, Blinder P, Friedman B, Drew PJ, et al (2009) Correlations of neuronal and microvascular densities in murine cortex revealed by direct counting and colocalization of nuclei and vessels. J Neurosci 29: 14553–14570. 19923289 10.1523/JNEUROSCI.3287-09.2009 PMC4972024

[9] Gotz J, Ittner LM (2008) Animal models of Alzheimer's disease and frontotemporal dementia. Nat Rev Neurosci 9: 532–544. 18568014 10.1038/nrn2420

[10] Carmeliet P, Jain RK (2000) Angiogenesis in cancer and other diseases. Nature 407: 249–257. 11001068 10.1038/35025220

[11] Blinder P, Tsai PS, Kaufhold JP, Knutsen PM, Suhl H, et al (2013) The cortical angiome: an interconnected vascular network with noncolumnar patterns of blood flow. Nat Neurosci 16: 889–897. 23749145 10.1038/nn.3426 PMC4141079

[12] Heinzer S, Krucker T, Stampanoni M, Abela R, Meyer EP, et al (2006) Hierarchical microimaging for multiscale analysis of large vascular networks. Neuroimage 32: 626–636. 16697665 10.1016/j.neuroimage.2006.03.043

[13] Hashimoto H, Kusakabe M, Ishikawa H (2008) A novel method for three-dimensional observation of the vascular networks in the whole mouse brain. Microsc Res Tech 71: 51–59. 17868133 10.1002/jemt.20522

[14] Mayerich D, Kwon J, Sung C, Abbott L, Keyser J, et al (2011) Fast macro-scale transmission imaging of microvascular networks using KESM. Biomed Opt Express 2: 2888–2896. 22091443 10.1364/BOE.2.002888 PMC3191452

[15] Erturk A, Becker K, Jahrling N, Mauch CP, Hojer CD, et al (2012) Three-dimensional imaging of solvent-cleared organs using 3DISCO. Nat Protoc 7: 1983–1995. 23060243 10.1038/nprot.2012.119

[16] Jahrling N, Becker K, Dodt HU (2009) 3D-reconstruction of blood vessels by ultramicroscopy. Organogenesis 5: 145–148. 10.4161/org.5.4.10403 PMC2878751 20539742

[17] Krucker T, Lang A, Meyer EP (2006) New polyurethane-based material for vascular corrosion casting with improved physical and imaging characteristics. Microsc Res Tech 69: 138–147. 16456839 10.1002/jemt.20263

[18] Tata DA, Anderson BJ (2002) A new method for the investigation of capillary structure. J Neurosci Methods 113: 199–206. 11772441 10.1016/s0165-0270(01)00494-0

[19] Li A, Gong H, Zhang B, Wang Q, Yan C, et al (2010) Micro-optical sectioning tomography to obtain a high-resolution atlas of the mouse brain. Science 330: 1404–1408. 21051596 10.1126/science.1191776

[20] Yang XQ, Meng YZ, Luo QM, Gong H (2010) High resolution in vivo micro-CT with flat panel detector based on amorphous silicon. J X-Ray Sci Technol 18: 381–392. 10.3233/XST-2010-0269 21045275

[21] Zhang B, Li A, Yang Z, Wu J, Luo Q, et al (2011) Modified Golgi-Cox method for micrometer scale sectioning of the whole mouse brain. J Neurosci Methods 197: 1–5. 20959121 10.1016/j.jneumeth.2010.10.001

[22] Rodriguez A, Ehlenberger DB, Hof PR, Wearne SL (2006) Rayburst sampling, an algorithm for automated three-dimensional shape analysis from laser scanning microscopy images. Nat Protoc 1: 2152–2161. 17487207 10.1038/nprot.2006.313

[23] Chen S, Mohajerani MH, Xie Y, Murphy TH (2012) Optogenetic analysis of neuronal excitability during global ischemia reveals selective deficits in sensory processing following reperfusion in mouse cortex. J Neurosci 32: 13510–13519. 23015440 10.1523/JNEUROSCI.1439-12.2012 PMC6621379

[24] Dorr AE, Lerch JP, Spring S, Kabani N, Henkelman RM (2008) High resolution three-dimensional brain atlas using an average magnetic resonance image of 40 adult C57Bl/6J mice. Neuroimage 42: 60–69. 18502665 10.1016/j.neuroimage.2008.03.037

[25] Weber B, Keller AL, Reichold J, Logothetis NK (2008) The microvascular system of the striate and extrastriate visual cortex of the macaque. Cereb Cortex 18: 2318–2330. 18222935 10.1093/cercor/bhm259

[26] Harrison RV, Harel N, Panesar J, Mount RJ (2002) Blood capillary distribution correlates with hemodynamic-based functional imaging in cerebral cortex. Cereb Cortex 12: 225–233. 11839597 10.1093/cercor/12.3.225

