# Supplementary figures and images for: Indian-Ink Perfusion Based Method for Reconstructing Continuous Vascular Networks in Whole Mouse Brain
Source: PLoS One. 2014 Jan 30;9(1):e88067. doi: 10.1371/journal.pone.0088067 (PMC3907580; doi:10.1371/journal.pone.0088067)

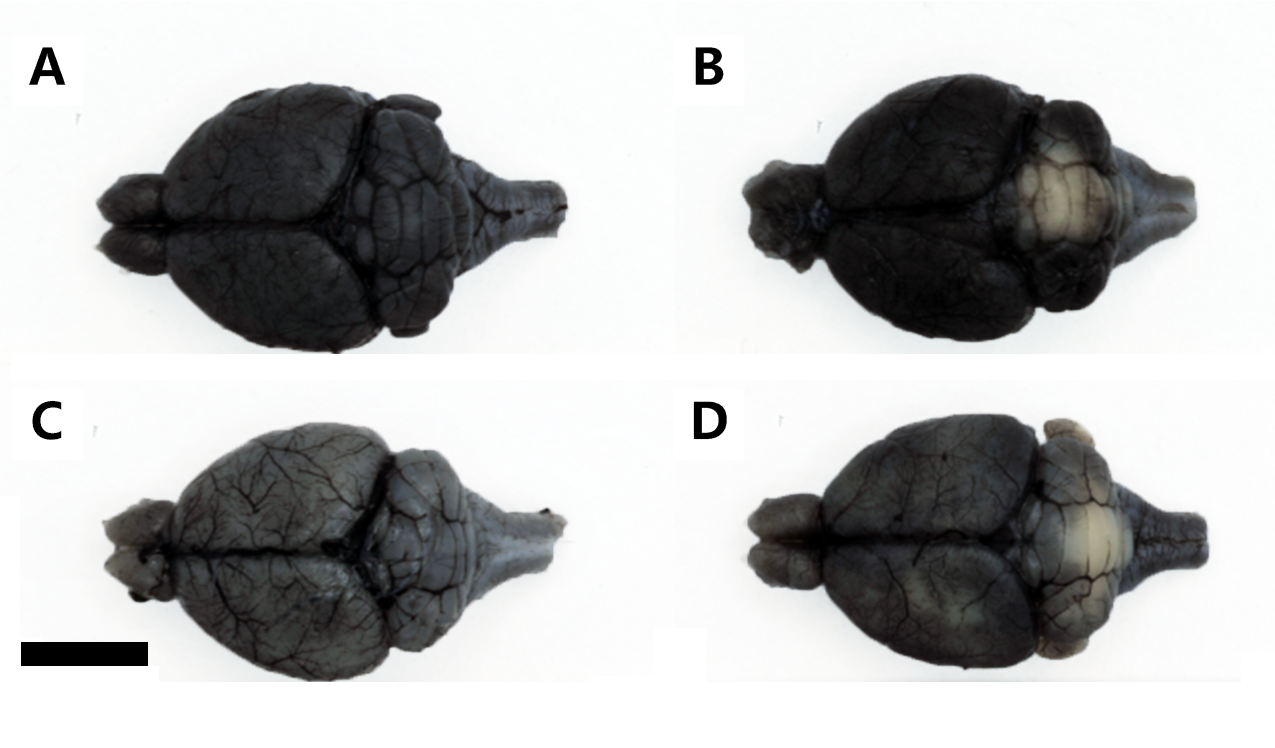

Supplement: Figure S1 — Visual inspection of Indian-ink perfused mouse brain. A: an effective Indian-ink perfused mouse brain shows uniform and black surface, which is used to get results in this paper; B: an incomplete Indian-ink perfused mouse brain, which is due to the high temperature and perfusion pressure; C: a uniform but incomplete perfused mouse brain, due to the low temperature and perfusion pressure; D: an un-uniform and incomplete perfused mouse brain, due to the impurities in perfusion fluid. Scale bar = 5 mm. (TIF) [file pone.0088067.s001.tif]

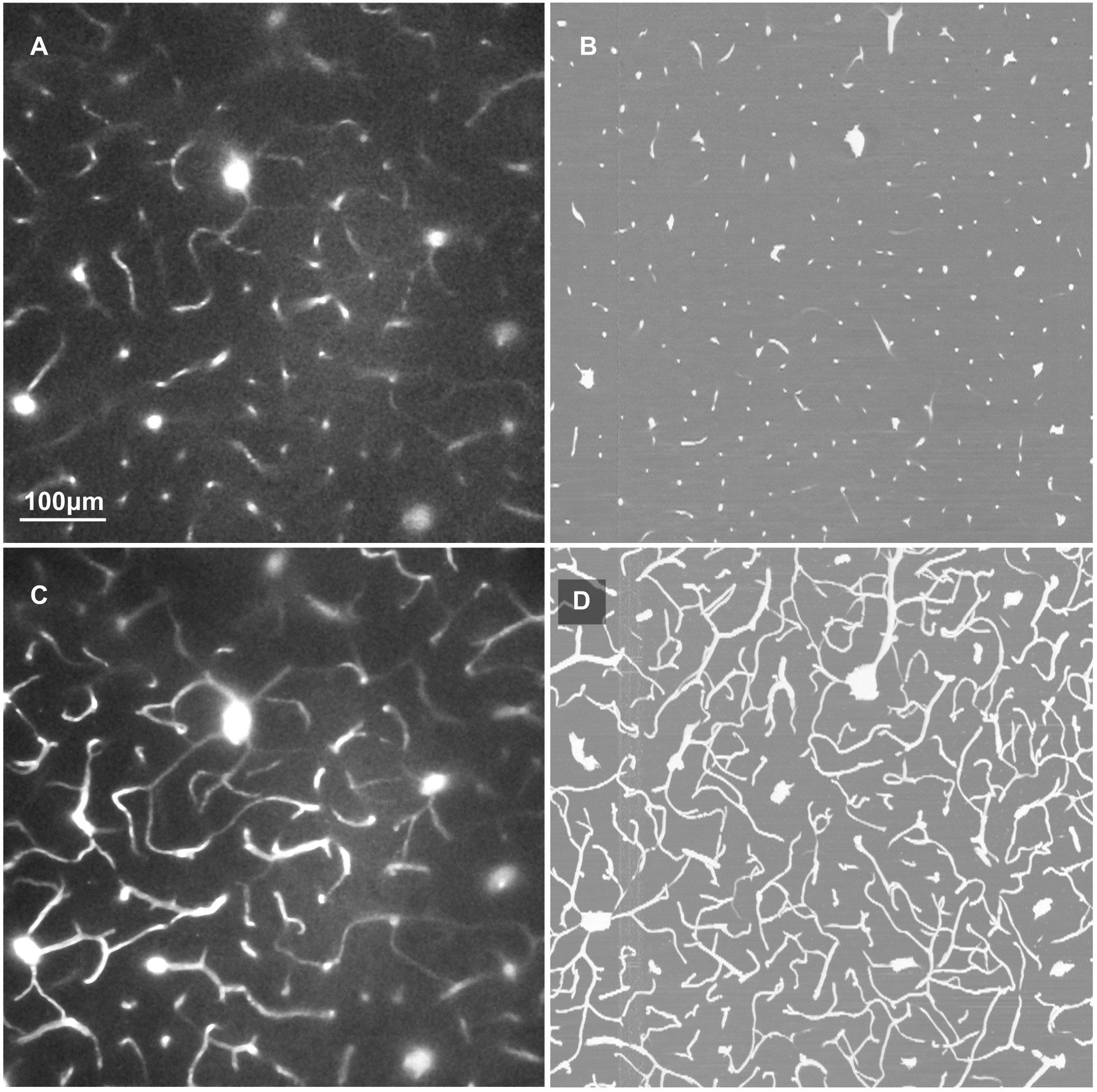

Supplement: Figure S2 — Comparisons between MOST imaging and in vivo two-photon imaging on calibers measurement. A: a 2 µm thickness image frame from in vivo two-photon fluorescence imaging on barrel cortex in an anesthetized Kunming mouse to measure the capillaries' calibers; B: a 2 µm thickness image frame from the MOST dataset (with intensity reverting and cut from the same dataset acquired in this study); C: 100 µm depth MIP reconstruction of two-photon vascular imaging; D: 100 µm depth MIP reconstruction of MOST vascular imaging. By randomly selecting 30 loci to measure the capillaries calibers in C and D, the two measurements showed consistent results. (TIF) [file pone.0088067.s002.tif]

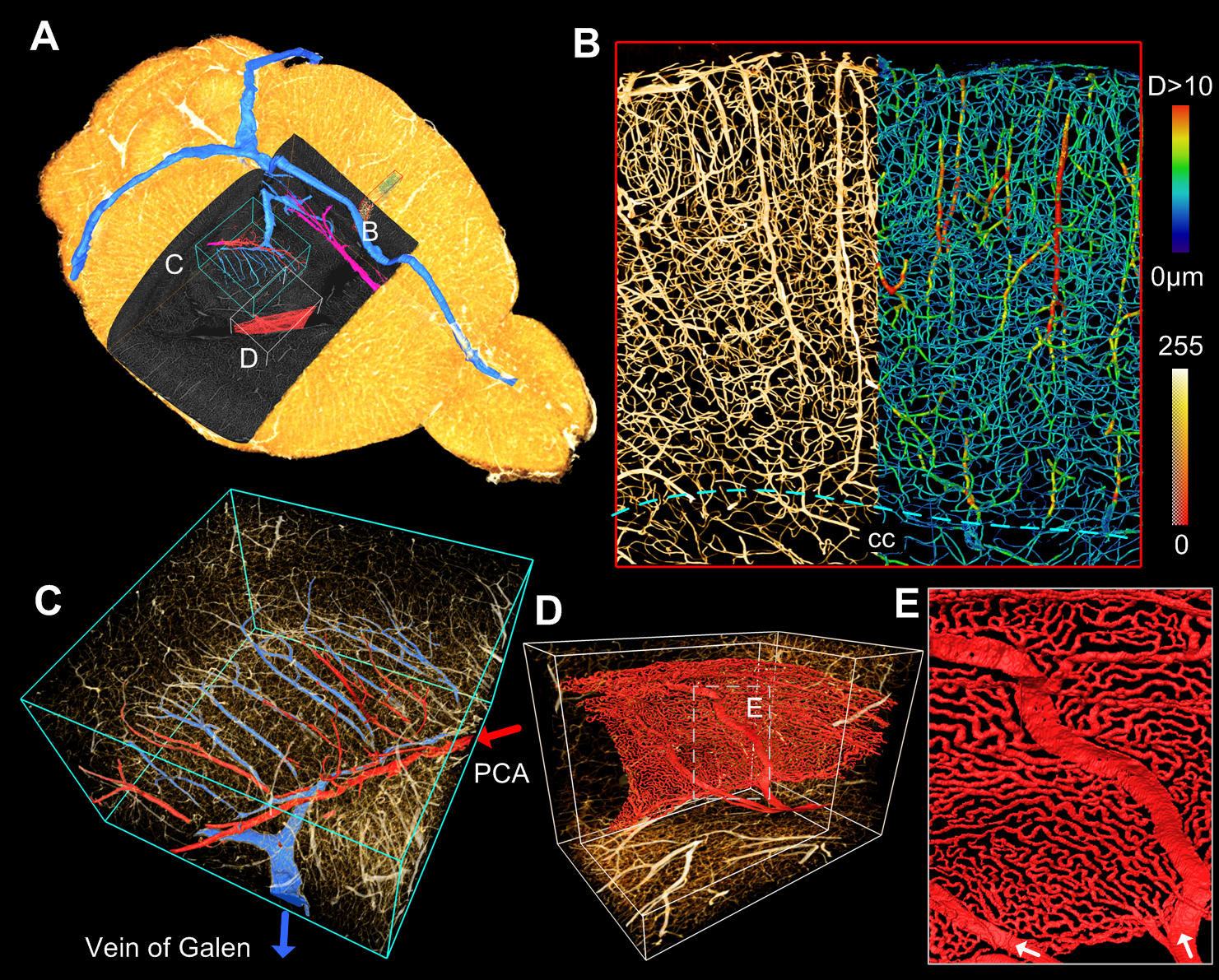

Supplement: Figure S3 — Vessel reconstruction in the cortex, hippocampus and choroid. A: The locations of the three ROIs. B: Vessel reconstruction and diameter tracking in the cortex using Amira, 1.0×1.0×0.2 mm3. C: Vessel reconstruction in the hippocampus, 1.5×1.0×1.5 mm3, red for arteries and blue for veins. D: Vessel surface reconstruction of the choroid in the lateral ventricle, 1.0×1.5×1.0 mm3; the arrows show the direction of the blood flow. (TIF) [file pone.0088067.s003.tif]

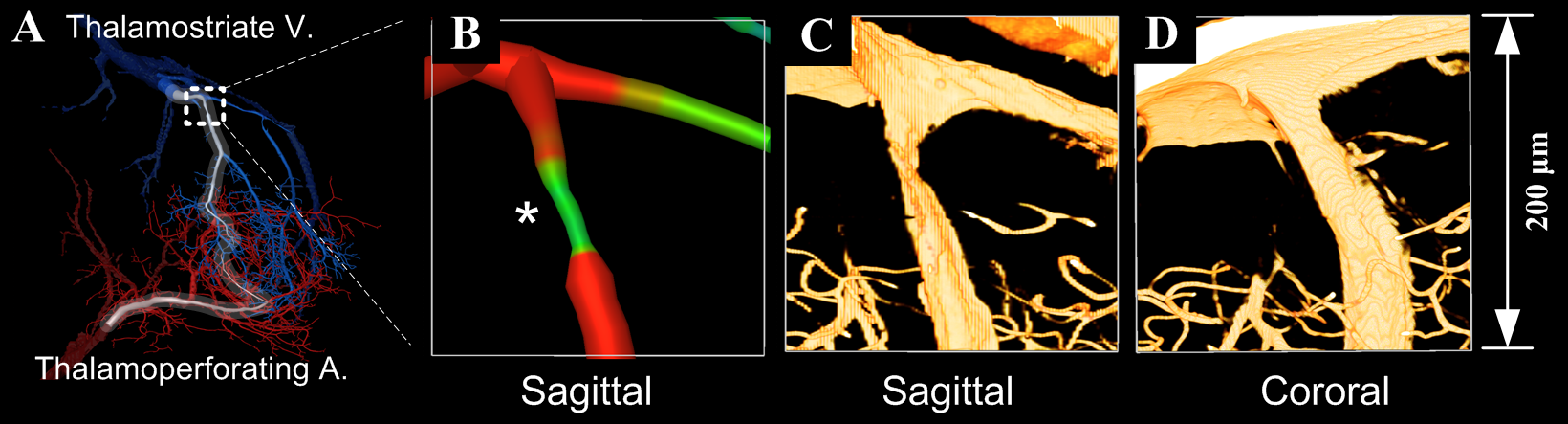

Supplement: Figure S4 — The RAW reconstruction of the vessel narrowing site which showed in Figure 3 . A B C, the right view; D the rear view; C D, volume rendering reconstruction of the vessels around the narrowing site. (TIF) [file pone.0088067.s004.tif]
